# Canopy Definitions Shape Canopy Space Filling–Productivity Relationships: Evidence From Terrestrial Laser Scanning

**DOI:** 10.1002/ece3.73610

**Published:** 2026-05-04

**Authors:** Tama Ray, Andreas Fichtner, Matthias Kunz, Michaela Hildebrand, Helge Bruelheide, Catherine Potvin, Florian Schnabel, Goddert von Oheimb

**Affiliations:** ^1^ Institute of General Ecology and Environmental Protection TUD Dresden University of Technology Tharandt Germany; ^2^ German Centre for Integrative Biodiversity Research (iDiv) Halle‐Jena‐Leipzig Leipzig Germany; ^3^ Institute of Biology/Geobotany and Botanical Garden Martin Luther University Halle‐Wittenberg Halle (Saale) Germany; ^4^ Institute of Ecology Leuphana University of Lüneburg Lüneburg Germany; ^5^ GFZ Helmholtz Centre for Geosciences Potsdam Germany; ^6^ ThüringenForst, Forestry Research and Competence Centre Gotha Germany; ^7^ Department of Biology McGill University Montréal Québec Canada; ^8^ Smithsonian Tropical Research Institute Panama Panama; ^9^ Chair of Silviculture Faculty of Environment and Natural Resources Freiburg Germany

**Keywords:** biodiversity, canopy space definition, canopy space filling index, Sardinilla BEF experiment, stand productivity, voxelisation

## Abstract

Many important processes in forest ecosystems are influenced by the spatial structure of the canopy. The spatial arrangement of trees and their branches within the canopy is crucial for light interception, often resulting in a positive relationship between canopy space filling and stand productivity. To date, there is no universal definition of canopy space. However, different parts of the canopy have distinct characteristics, making it crucial to assess the significance of varying canopy definitions. In this study, we investigated how canopy space filling changes with four different definitions of canopy space by using a canopy space filling index (CSFI) derived from terrestrial laser scanning. Moreover, we assessed the relative importance of these definitions in explaining stand productivity. Using data from a tropical tree diversity experiment, we found that CSFI strongly varied with the specific delineation of the canopy space. Across canopy definitions, stand productivity was significantly and positively affected by CSFI, indicating that this relationship was generally robust to varying definitions of canopy space. However, excluding the uppermost part and including the lowest part of the canopy space resulted in a distinct decline of explained variance in productivity. In addition, CSFI played a more important role in regulating productivity in mixtures compared to monocultures. Our results highlight the importance of different layers of the canopy space and tree species richness for a better understanding and assessment of forest dynamics and ecosystem functions. Further research is needed on the relationship between canopy space filling and stand productivity based on different canopy space definitions for mature forests and from different biomes.

## Graphical abstract

The spatial arrangement of trees and their branches within the canopy is crucial for light interception, often resulting in a positive relationship between canopy space filling and stand productivity. To date, there is no universal definition of canopy space. However, different parts of the canopy have distinct characteristics, making it crucial to assess the significance of varying canopy definitions. Therefore, our study explained how stand productivity was significantly and positively affected by canopy space filling index across canopy definitions.

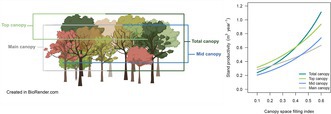

## 1 Introduction

Tree crowns and their structural characteristics play a crucial role in providing niche opportunities for biodiversity of other trophic levels and supporting ecosystem functioning of forest ecosystems (Ozanne et al. 2003). As an important component of forest community, trees are involved in neighbourhood interactions that shape their particular architectural stature (Fichtner et al. 2017; von Oheimb et al. 2011; Trogisch et al. 2021). In their entirety, individual tree crowns form the canopy, which makes a central contribution to key processes such as community‐level photosynthesis and transpiration, thereby significantly influencing the growth and productivity as well as biomass allocation of forests (Antonarakis et al. 2011; Fichtner et al. 2017; Gough et al. 2019; Potvin and Dutilleul 2009; Pretzsch 2014). In addition, canopy dynamics have a profound impact on forest biomass accumulation and carbon storage (Schnabel et al. 2025; Lefsky et al. 2002; Saatchi et al. 2011; Zhang et al. 2014).

Several studies from forest biodiversity—ecosystem functioning (BEF) experiments have shown that tree species richness strongly determines forest productivity (Huang et al. 2018; Ray et al. 2023). Specifically, spatial complementarity among trees in species mixtures enhanced canopy space filling, thereby increasing stand productivity (Ray et al. 2024). Canopy space filling (CSF; Juchheim et al. 2017; Pretzsch 2014), also known as canopy space occupation (Georgi et al. 2022) or canopy packing (Jucker et al. 2015), refers to the amount of aboveground space occupied by tree components (Juchheim et al. 2017; Pretzsch 2014). There is, however, no simple definition of what ‘canopy space’ exactly is. For technical reasons, it has often been defined as the space between the highest tree top and the lowest green branch of the total area included in a study. In this way, the canopy space is expressed as the maximum volume of space that the forest type under study has developed. Based on this single maximum value for the canopy space, the CSF at plot level can be given either as an absolute value (absolute CSF) or as a fraction of the maximum occupied voxels out of the total voxelised canopy space (i.e., a voxel is a volumetric element shaped as a cube), hereafter referred as canopy space filling index (CSFI).

**FIGURE 1:**
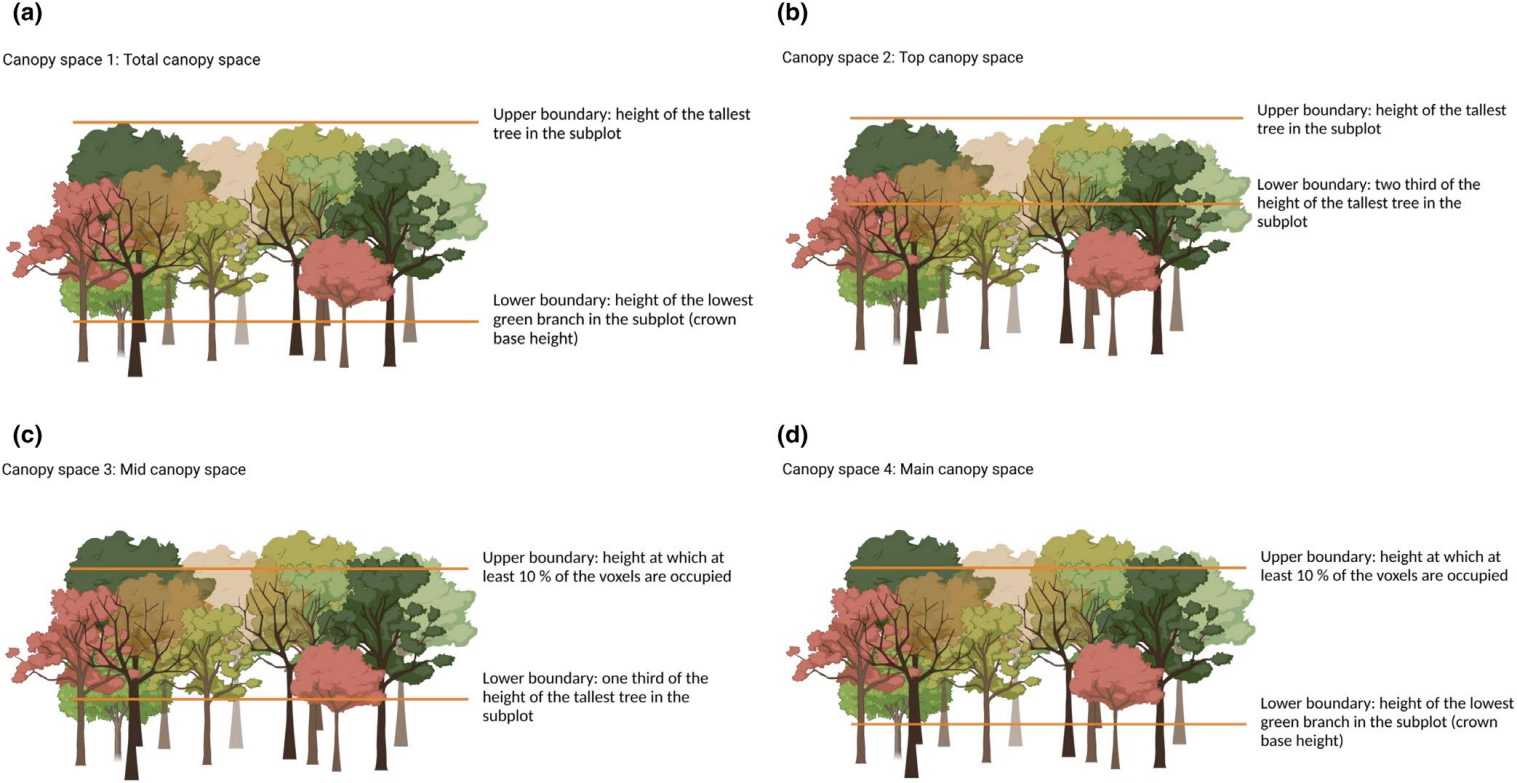
Definitions of the canopy space based on different upper and lower boundaries. Upper and lower lines denote the upper and lower boundary of the canopy, respectively (Created in Biorender.com).

This approach is conceptually simple but has limitations, particularly because a single maximum canopy volume may not adequately capture spatial heterogeneity within and among plots (Georgi et al. 2022). To overcome these limitations, alternative approaches define canopy for finer spatial scales or for different canopy regions (Figure 1), using plot‐specific metrics such as the maximum tree height and the lowest green branch. To account for differences in canopy volume among study plots, it is essential to use relative measures, such as CSFI.

Accurate quantification of CSFI requires detailed information on three‐dimensional canopy structure. Light detection and ranging (LiDAR) is a modern surveying technology that provides a robust method for analysing three‐dimensional (3D) occupancy in canopy space, particularly when the voxelisation (or voxel grid) approach is used (Georgi et al. 2022; Pearse et al. 2019; Seidel et al. 2013). In this approach, 3D point clouds are subdivided into voxels of defined size and the proportion of empty and occupied voxels is used to quantify CSFI (see Hess et al. 2018; Seidel et al. 2013 for details). This enables a high‐resolution characterisation of canopy structure, which is essential for understanding the relationship between canopy space filling and biomass productivity. Such detailed structural information is particularly important in mixed‐species stands, where diversity‐driven tree–tree interactions influence crown size and shape and, consequently, canopy architecture (Guillemot et al. 2020; Hildebrand et al. 2021; Kunz et al. 2019; Sapijanskas et al. 2014). Using this voxel‐based approach, Seidel et al. (2013) and Georgi et al. (2022) further separated canopy space into different height strata and demonstrated that the effects of tree species richness on canopy space filling vary across these strata.

A definition based on the canopy space between the highest tree height and the crown base may, however, not always be sufficient to fully capture the structural diversity and dynamics of forests, as these vary greatly depending on management practices (Stiers et al. 2018) and tree species richness (Perles‐Garcia et al. 2022). For example, many primaeval forests have a complex structure due to vertical stratification resulting from natural ageing and regeneration processes (Willim et al. 2022). Managed forests of uneven age can achieve a high structural complexity because silvicultural interventions aim at promoting different tree sizes (Ehbrecht et al. 2017). High management intensity with even‐aged trees often results in a simpler forest structure. In contrast, mixed‐species stands show greater vertical stratification due to the different functional characteristics of the tree species and higher diversity‐induced individual tree plasticity (Jucker et al. 2015; Kunz et al. 2019). For example, light‐demanding trees interact for light and resources in the upper canopy, while shade‐tolerant species in the mid and lower canopy layers are adapted to low‐light conditions. All this leads to differences in canopy space utilisation, particularly among different height layers. To account for such variability, Seidel et al. (2013) proposed subdividing the canopy based on height alone. However, this approach may not fully capture canopy space differentiation across different forest dynamics and types. Georgi et al. (2022) redefined canopy space definitions by adjusting upper and lower boundaries. In our study, we adopt this approach, but extend it in several important ways: First, we apply it to a tree diversity experiment with strictly controlled tree species composition and richness, rather than a managed production forest. Second, we used a modified set of canopy space definitions. Finally, we incorporate tree growth data to analyse how different canopy space definitions affect the relationship between CSFI and stand productivity. The four canopy space definitions used in this study are illustrated in Figure 1: (a) *Total canopy space*, extending from the highest tree top to the height of the lowest green branch in the subplot (Georgi et al. 2022); (b) *Top canopy space*, extending from the highest tree top to two‐thirds of the total height in the subplot (Georgi et al. 2022); (c) *Mid canopy space*, extending from the height where at least 10% of the voxels were occupied to one third of the total height in the subplot; (d) *Main canopy space*, extending from the height where at least 10% of the voxels were occupied to the height of the lowest green branch in the subplot.

In this study, we used the Sardinilla tree diversity‐ecosystem functioning (BEF) experiment in Panama and created a high‐resolution 3D dataset using terrestrial laser scanning (TLS) to obtain detailed information about the structure of the canopy space. In monoculture and mixed‐species plots we quantified the canopy space filling based on the four different canopy space definitions explained above (Figure 1) and related them to stand productivity. Specifically, we investigated (1) how different canopy space definitions affected CSFI and (2) whether canopy space definitions or mixture type (i.e., monocultures versus tree species‐mixtures) modified the relationship between CSFI and stand productivity. We hypothesised that (H1) the mid canopy space (Figure 1c) has a higher CSFI than the other canopy space definitions and (H2) mid canopy space displays the strongest relationship with stand productivity. Given that stand productivity is significantly enhanced by a higher degree of canopy space filling (Ray et al. 2024), we assumed that only the densely filled parts of the canopy contribute to the productivity of the stand.

## 2 Methodology

### 2.1 Study Site

The research was conducted in the tropical BEF experiment located in Sardinilla, Panama (9°19′ N, 79°38′ W) (https://treedivnet.ugent.be/). The climatic conditions of the site are characterised by an average annual temperature of 26°C and an annual rainfall of 2661 mm (BCI, Physical Monitoring Program, Smithsonian Tropical Research Institute). The area was covered by native semi‐deciduous lowland forests that were cleared in 1952/1953. The 5 ha area was subsequently used for agriculture before being converted into pasture (Scherer‐Lorenzen et al. 2005).

In 2001, 5566 seedlings of six native tree species under six months old were planted at 3 m intervals using standard Panamanian reforestation practices (Potvin and Gotelli 2008; Potvin and Dutilleul 2009), with seedlings arranged in monocultures, three‐species mixtures and six‐species mixtures (Potvin and Dutilleul 2009). The species included fast‐growing pioneer species: *Luehea seemannii* (Malvaceae) and *Cordia alliodora* (Boraginaceae); light‐intermediate species: *Anacardium excelsum* (Anacardiaceae) and *Hura crepitans* (Euphorbiaceae); and slow‐growing shade‐tolerant species: *Cedrela odorata* (Meliaceae) and *Tabebuia rosea* (Bignoniaceae). The experiment was arranged across 24 plots (45 × 45 m each), of which 12 monocultures, six three‐species mixtures and six six‐species mixtures (Potvin and Dutilleul 2009; Scherer‐Lorenzen et al. 2005). Each plot was further divided into four subplots of equal size. Three‐species mixtures were designed for trait divergence, with species selected based on growth rates and ecological roles at Barro Colorado Island (Scherer‐Lorenzen et al. 2005). Each mixture of three species included a fast‐growing pioneer species, a light‐intermediate species and a slow‐growing shade‐tolerant species. Due to high mortality of *C. alliodora* (> 85%) in the early years (Potvin and Gotelli 2008; Scherer‐Lorenzen et al. 2007), monoculture data of this species is unavailable. However, the mortality rate of the subplots was relatively low between 2012 and 2017. This resulted in a total of 22 plots and 88 subplots.

### 2.2 TLS Data Acquisition and Post‐Processing

Data from TLS was acquired in early June 2017 with a RIEGL VZ‐400i scanner (RIEGL, Austria). The scanner provided a 360° horizontal and 130° vertical field‐of‐view with a capacity of full‐waveform analysis. Every plot was scanned in multi scan mode at 16 scanning positions, with two scans at each scan position. The first scan was at the vertical position, and the second one was at the horizontal position with a 90° tilt, which provides a complete spherical view of the canopy space to conduct full waveform analysis. The laser scanner uses a 1550 nm wavelength and features an angular resolution of 0.04°, resulting in a point sampling interval of approximately 7 mm at a distance of 10 m. To enhance canopy penetration, the scanning frequency was configured to operate at 600 kHz (Guillemot et al. 2020). To address scan shadows, scan positions were spaced *ca*. 15 m apart in a systematic pattern, ensuring each tree was captured from multiple angles to represent 3D structures. Scanning operations took place under dry and stable weather conditions, with calm winds and temperatures averaging 25°C (for more details, see Guillemot et al. 2020).

Point clouds were co‐registered in RiSCAN Pro (v2.6.2) using multi‐station adjustment with plane patches, achieving < 3 mm accuracy (Guillemot et al. 2020). A 2 m buffer area at the boundary of each plot was removed to reduce the edge effects, and the resulting plot was divided into four subplots. The plot point clouds were cropped with lasclip (LAStools 2022).

### 2.3 Canopy Space Filling Analysis

The interior of the clipped area was divided into voxels, each measuring 40 cm edge length to capture ecologically meaningful canopy variation while mitigating occlusion effects. This method, known as the ‘voxel model,’ has been widely utilised in various applications, including TLS (e.g., Hosoi and Omasa 2007), airborne laser scanning (e.g., Chasmer et al. 2004) and canopy modelling (e.g., Myneni et al. 1997; Sinoquet et al. 2001). The canopy space filling was analysed for each subplot following the method described by Georgi et al. (2022). The canopy space within each subplot was voxelised using lasvoxel (LAStools 2022) minimising occlusion effects in tropical trees with full foliage, as suggested by Béland et al. (2011). For each subplot and canopy space definition, we calculated a CSFI as the fraction of occupied voxels within the total voxelised canopy space (Figure S2).

### 2.4 Stand Productivity

The diameter at breast height (D) and the height (H) per tree in every subplot were measured yearly from 2012 to 2017. We estimated wood volume (*V*_*i*_, m^3^), for each tree *i*, using the formula $V_{i}=\left(\pi D_{i}^{2}/4\right)H_{i}f$. To consider the cylinder shape of a tree, a form factor of 0.4 was applied (Kunz et al. 2019; Pretzsch 2009).

The wood volume *V*_*ij,1*_ of tree *i* within subplot *j* was calculated at the beginning of the study (*t*_1_) in 2012, while *V*_*ij,2*_ denotes the wood volume of the same tree at the end of the study (*t*_2_) in 2017. Therefore, annual wood productivity (AWP, cm^3^ year^−1^) for each subplot *j* was calculated as the sum of the annual growth rates of all living trees (*n*) within a subplot:

$${\mathrm{AWP}}_{j}=\sum_{i=1}^{n}\left(\frac{V_{\mathrm{ij},2}-V_{\mathrm{ij},1}}{t_{2}-t_{1}}\right)$$

### 2.5 Statistical Analysis

**FIGURE 2:**
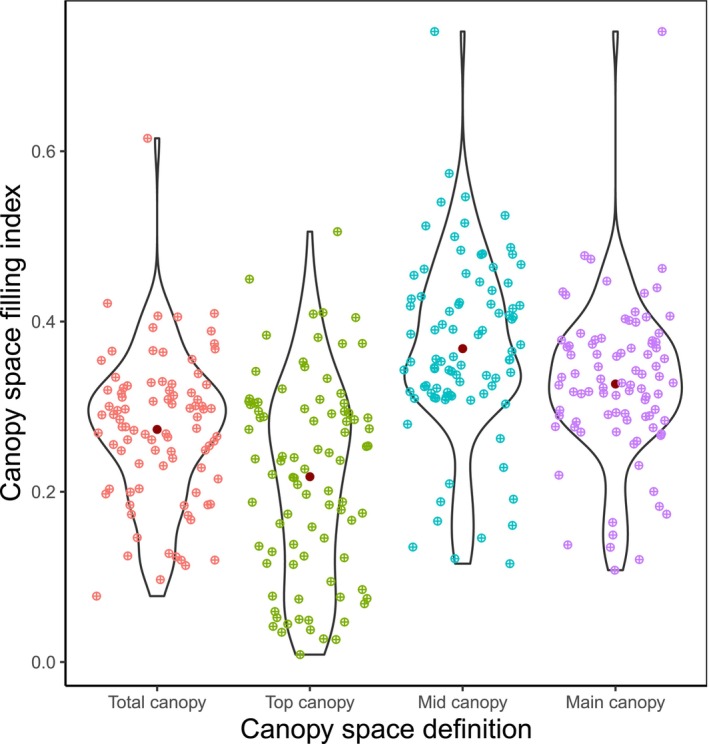
Changes in canopy space filling index (CSFI) with canopy space definition. Violin plots show the distribution of CSFI across different canopy space definitions. The mean value for each group is marked in dark red, with individual data points are coloured to display their distribution within each category.

We used generalised linear mixed‐effect models (GLMMs) to examine how the relationship between stand productivity and CSFI vary in strength and direction with canopy space definition. To avoid biases associated with logarithmic transformations, we employed a gamma probability distribution with a log‐link function to ensure homoscedasticity of the residuals (Zuur et al. 2009). Given that each plot was divided into four subplots, plot identity was included as a random effect to account for the nested structure of subplots within plots. This approach also accounts for the realised compositional differences in tree species among subplots. Due to collinearity between CSFI and canopy definition (Figure 2), we fitted the GLMMs for each canopy definition separately. Moreover, we tested if the magnitude of CSFI effect on AWP varies with mixture type by including an interaction term between CSFI and mixture type in the GLMMs. Mixture type was fitted as a two‐level factor in the models (i.e., monocultures and mixed‐species subplots). All analyses were performed in R (version 4.3.3; R Core Team 2024) using the packages lme4 (Bates et al. 2015), lmerTest (Kuznetsova et al. 2017), MuMIn (Bartoń 2023), viridis (Garnier et al. 2024), glmmTMB (Brooks et al. 2017), dplyr (Wickham et al. 2023) and gridExtra (Auguie and Antonov 2017).

## 3 Results

### 3.1 Effects of Canopy Space Definition on Canopy Space Filling

The CSFI strongly varied with canopy space definition (Figure 2). The CSFI was highest considering the mid and main canopy space, while considering the top canopy space only resulted in the lowest CSFI values. For example, the CSFI was on average 35% and 69% higher in mid canopy space compared to total and top canopy space, respectively. The CSFI of all four definitions was closely and positively correlated (all Pearson's correlation coefficients *r* > 0.64; Figure S1). The total canopy space, the mid canopy space and the main canopy space were particularly strongly correlated (*r* > 0.90). The correlation between the top canopy space and the other three canopy space definitions was less pronounced with the strongest correlation between top and total canopy space.

### 3.2 Effects of Canopy Space Definition on Canopy Space Filling—Productivity Relationships

**FIGURE 3:**
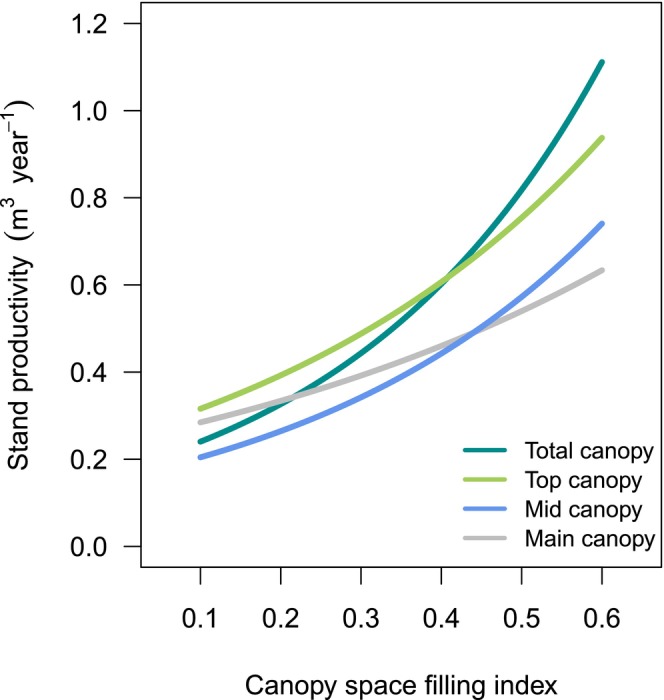
Relationship between canopy space filling index (CSFI) and stand productivity (AWP). Regression lines correspond to the predicted response of generalised mixed‐effect models, using the common observed CSFI range. Models were fitted for each canopy space definition separately.

**FIGURE 4:**
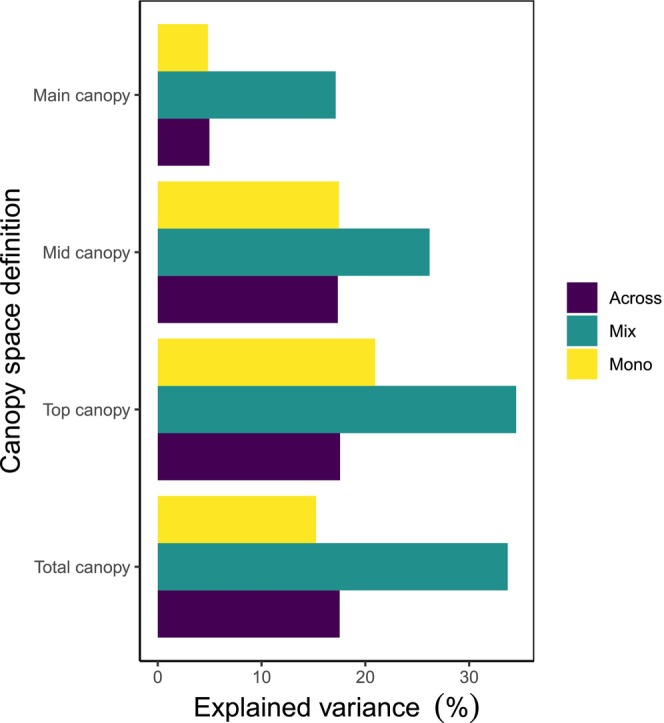
Changes in explained variance in stand productivity (annual wood productivity, AWP) by different canopy definitions. Different colours indicate marginal *R* ^2^‐values of generalised mixed‐effect models that were fitted across monocultures and mixtures (purple) and for monocultures (yellow) and mixtures (dark cyan) separately.

Across canopy space definitions, AWP significantly increased with CSFI (Table 1). However, the strength of this relationship strongly varied with canopy space definition. Total canopy showed the strongest and main canopy the weakest increase in annual wood productivity (AWP) with CSFI, respectively. The magnitude of the CSFI‐AWP relationship was similar for top and mid canopy (Figure 3). Across monocultures and mixtures, the CSFI explained between 17.5% (total, top, mid canopy) and 5% (main canopy) of the variation in AWP (Figure 4).

**TABLE 1:** Results of generalised mixed‐effect models for the effect of canopy space filling index (CSFI) on stand productivity (annual wood productivity, AWP).

Definition	Mix types		Estimate	SE	*p*
Total canopy	Across	CSFI	3.0635	0.8905	**< 0.001**
	Mixtures	CSFI	4.2983	1.0989	**< 0.001**
	Monocultures	CSFI	2.5244	0.9989	**< 0.05**
Top canopy	Across	CSFI	2.1767	0.5377	**< 0.001**
	Mixtures	CSFI	2.6841	0.682	**< 0.001**
	Monocultures	CSFI	2.1697	0.6832	**< 0.05**
Mid canopy	Across	CSFI	2.5755	0.7225	**< 0.001**
	Mixtures	CSFI	3.1546	0.9629	**< 0.05**
	Monocultures	CSFI	2.3185	0.889	**< 0.01**
Main canopy	Across	CSFI	1.6012	0.7944	**< 0.001**
	Mixtures	CSFI	3.0637	1.2541	**< 0.05**
	Monocultures	CSFI	1.3565	0.8776	0.122

*Note:* For each canopy space definition, models were fitted across monocultures and mixtures (across) and for monocultures and mixtures separately. Significant relationships (*p* < 0.05) are highlighted in bold.

**FIGURE 5:**
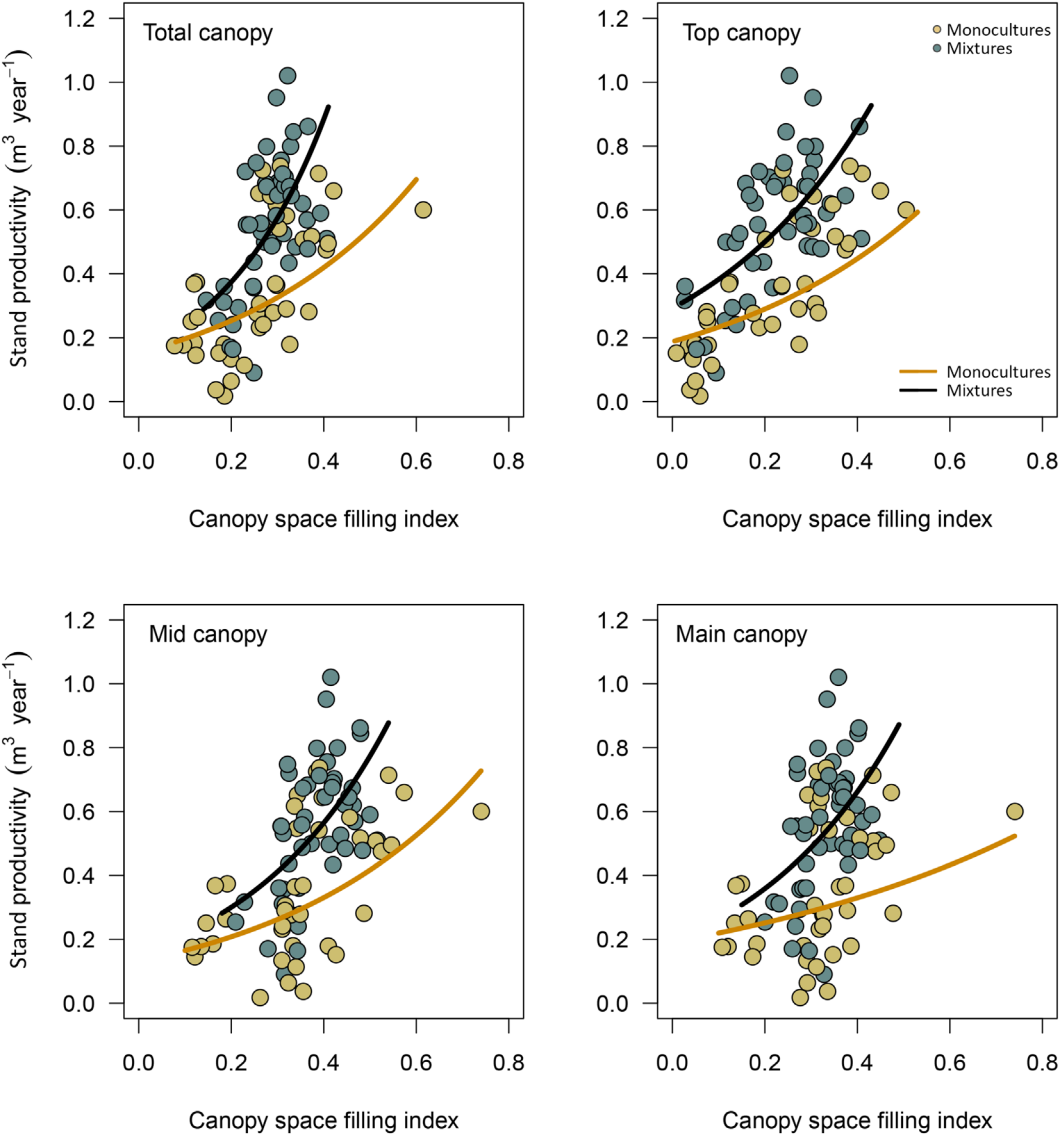
Relationship between canopy space filling index (CSFI) and stand productivity (AWP) in monocultures and tree species mixtures for different canopy space definitions. Regression lines correspond to the predicted response of generalised mixed‐effect models fitted for monocultures (black) and mixtures (dark orange) separately. Note that for each canopy definition the interaction between CSFI and mixture type (i.e., monocultures and mixtures) was statistically not significant. Points represent CSFI and AWP values measured in each subplot, while the colour of the points corresponds to observed values in monocultures (light brown) and mixed‐species subplots (dark cyan).

For each canopy definition, AWP increased with CSFI in both monocultures and mixtures (Figure 5), although this relationship was not statistically significant for monocultures under the Main canopy definition (Table 1). The strength of the CSFI effect on AWP, however, was not dependent on mixture type (i.e., monocultures and mixtures), as indicated by the non‐significant interaction between CSFI and mixture type (total canopy: *p* = 0.741; top canopy: *p* = 0.435; main canopy: *p* = 0.763; mid canopy: *p* = 0.824; Table S1). In contrast, the relative importance of CSFI for AWP (expressed by the explained variance in AWP by CSFI; marginal *R* ^2^‐value) was distinctly higher for mixtures compared to monocultures (Figure 4). This pattern was consistent across canopy space definitions. For top and total canopy, the CSFI explained 35% (top canopy) and 34% (total canopy) of the variation in AWP in mixed‐species subplots and marginal *R* ^2^‐value were 0.6‐times (top canopy) to 1.2‐times (total canopy) higher in mixtures compared to monocultures. For mid and main canopy, marginal *R* ^2^‐values ranged between 0.262 (mid canopy) and 0.171 (main canopy) in mixtures, while monocultures exhibited values between 0.174 (mid canopy) and 0.048 (main canopy).

## 4 Discussion

Under the controlled conditions of one of the oldest forest biodiversity experiments, the Sardinilla experiment, we found that the specific delineation of the canopy space determines the magnitude of the values for CSFI. The relationship between CSFI and stand productivity was generally robust to the exact definition of canopy space, as CSFI almost always had a significant positive effect on productivity. However, the variance explained was higher in the mixtures than in the monocultures, indicating that tree species richness had a strong influence.

### 4.1 Effects of Canopy Space Definition on Canopy Space Filling

There is no universally accepted definition of the exact vertical extent of the forest canopy. In the past, for mainly practical reasons, the canopy has often been defined very broadly, extending from the height of the highest tree to the lowest green branch. At the subplot level, this corresponds to our definition of total canopy space. At the top of the canopy, however, canopy space filling is generally not as high. This is reflected in the difference between the two definitions, total and main canopy space: the mean CSFI increased from 0.27 to 0.33 if the lower boundary remained the same, but the upper boundary was lowered to the height where at least 10% of the voxels were occupied. Georgi et al. (2022) justified this lowering by stating that a minimum level of canopy space filling must be present for tree–tree interactions to be effective. As space filling was often lower in the lowest part of the canopy, we expected that raising the lower boundary from the lowest green branch to one third of the height of the tallest tree would also increase canopy space filling. This was the case when comparing the definitions of main and mid canopy space (mean CSFI 0.33 and 0.36, respectively). Our hypothesis H1, that among the four definitions the highest canopy space filling should be reported in the mid canopy space, was confirmed. However, an important result of our analyses was that despite these differences in the extent of CSFI, the values of the three definitions total, mid and main canopy space were highly positively correlated with each other. The by far lowest values for CSFI in the top canopy space definition confirmed the assumption that canopy space filling was generally more patchy in the top canopy space. At the same time, however, the much greater variability of the values in the 25% to 75% percentile range with this definition was also striking. In general, it can be concluded that the exact delineation of the canopy space is of great importance for the level of CSFI values, but that there are consistently very high correlations in the stands analysed. The latter can be explained by the relatively young age and relatively high stand density in these experimental plots. In the case of older stands, or the inclusion of stands managed at different intensities, the differences between the different definitions could be even greater and the correlations much less close.

Tree species richness and composition also have an important influence on canopy space filling, because tree species mixtures have a high potential to occupy the canopy space more efficiently. Our results with the highest CSFI in the mid canopy space and a high correlation with the other canopy definitions may also be due to the fact that we included both monocultures and mixtures, and trait divergence due to different growth rates and shade tolerances was present in all mixtures. With increasing tree species richness, crown dimension increases as a result of local neighbourhood interactions (Fichtner et al. 2018; Georgi et al. 2022). These architectural changes of the crowns are due to diversity‐induced plasticity, which considerably improves spatial adaptation within the canopy. However, phenotypic adaptation in mixtures occurs not only via the outer crown structure, through changes in crown size and shape, but also via the inner crown structure, through changes in branch density and branching intensity. In this way, better spatial adaptation can be achieved even with constant canopy dimensions through greater plasticity at the branch level (Hildebrand et al. 2021). Mixtures also increase the evenness of biomass distribution at different height strata. Mixtures of shade‐intolerant and shade‐tolerant tree species, that is functionally dissimilar species in terms of their light requirements, grow in the upper and lower layers, respectively (Jucker et al. 2015). In addition, the shade‐tolerant species tend to have larger crowns (Poorter et al. 2006). In the mixture, these trees therefore invest more in vertical space exploration than in height growth (the slenderness ratio is lower in the mixture; del Río et al. 2019; Martin‐Ducup et al. 2016), resulting in a more evenly filled canopy space than in monocultures.

### 4.2 Effects of Canopy Space Definition on Canopy Space Filling—Productivity Relationships

Our results showed that stand productivity increased with increasing CSFI for all four definitions of canopy space. This confirms the findings of Ray et al. (2024) from the Sardinilla experiment that communities with high canopy space filling had 61% higher productivity than communities with low canopy space filling and highlights the importance of canopy space filling for productivity (Guillemot et al. 2020; Juchheim et al. 2017).

Three results from our analyses are particularly noteworthy: Firstly, the three definitions total, top and mid canopy space explained very similar proportions of the variance in the relationship between CSFI and stand productivity when all subplots (across) were included, whereas the main canopy space definition explained a much lower proportion of the variance. Secondly, the top canopy space definition explained a high proportion of the variance, even though CSFI values were lowest among all four definitions. And thirdly, we found very large differences between the monocultures and the mixtures in all four canopy space definitions, with the total and top canopy space being very similar, while the main canopy space, in comparison, was characterised by very low proportions of explained variance in the relationship between CSFI and stand productivity.

Our second hypothesis (H2) that mid canopy space displays the strongest relationship with stand productivity was only partly confirmed, because total, top and mid canopy space did not differ in the strength of the relationship. By contrast, the main canopy space explained much less variation in stand productivity, even though this definition had the second highest CSFI and was very strongly correlated with total and mid canopy space. Due to the differences in the respective definitions, we can conclude that both the upper and the lower canopy layer are very important predictors of stand productivity, but in opposite directions: with the identical lower canopy layer boundary to the lowest green branch, the inclusion of the uppermost canopy layer in the total canopy space definition led to a very large increase in explained variance compared to its omission in the main canopy space definition. With an identical upper limit of the canopy layer to at least 10% occupied voxels, omitting the lowest canopy layer in the mid canopy space definition led to a very large increase in explained variance compared to including it in the main canopy space definition.

Capturing the structural variation in the upper canopy seems to be of high importance, probably because in this layer most of the radiation energy conversion takes place, which in turn, affects photosynthesis and overall productivity. The canopy space assessment was based on TLS, which may be affected by vegetation‐induced occlusion (Wilkes et al. 2017). In dense stands, lower canopy elements can partially block the laser beam, potentially leading to an underrepresentation of upper canopy structures (Calders et al. 2020). However, since all plots were scanned using the same TLS protocol, any occlusion‐related bias is likely consistent across plots and should therefore not strongly influence relative comparisons (Wilkes et al. 2017). Shade tolerant species dominate the lower layer in functionally diversified mixed species stands (Niinemets 2010). Horizontal expansion leads to filling small gaps (Feldmann et al. 2018), particularly by first growing light demanding species, while larger gaps were filled mainly by vertical growth from lower canopy layers (Feldmann et al. 2018). There is strong evidence that the overall plasticity is higher in shade intolerant species while traits for example leaf morphology and nitrogen content are adapted to enhance light capture efficiency (Niinemets and Valladares 2004; Portsmuth and Niinemets 2007). Therefore, in functionally diversified mixed‐species stands, both light‐demanding and shade‐tolerant species benefit from a well‐structured canopy through complementarity of light use by reducing competition and optimising light distribution throughout the canopy (Ray et al. 2023). Therefore, it is likely the reason we found the strong correlation of CSFI with productivity in mixed stands. In competitive environments, especially where light is a limited resource, biomass is often allocated to height growth due to size‐asymmetric competition (Falster and Westoby 2003; Pretzsch 2009). However, in mixed stands, competitive reduction is the main driver of positive diversity effects, leading to more balanced resource use (Fichtner et al. 2017). This can occur horizontally through growth into canopy gaps or vertically through the formation of a lower crown base, that is deeper crowns, for shade‐tolerant species and increased height growth in the upper canopy for shade‐intolerant species (Potvin and Dutilleul 2009). As a result, mixtures develop higher leaf area indices than monocultures, as more leaf layers extend from top to bottom (Schnabel et al. 2025; Morin et al. 2011).

## 5 Conclusion

Our results show a positive relationship between canopy space filling and stand productivity across the different canopy space definitions. However, the strength of this relationship varied among canopy definitions but not between mixture types (i.e., monocultures versus tree species‐mixtures). As the explained variance of this relationship differs considerably between the definitions, total canopy space can be used for further CSFI analysis as it covers the entire canopy layers and appears to be a good predictor of stand productivity. Furthermore, the definition and measurement of total canopy space is relatively straightforward using both traditional and remotely sensed measurements. However, our study is based on a young, tropical experimental site, thus, more studies are required in mature forests and different biomes which could lead to a more adopted definition of the canopy space.

## Author Contributions

**Tama Ray:** conceptualization (equal), data curation (equal), formal analysis (equal), investigation (equal), methodology (equal), software (equal), visualisation (equal), writing – original draft (equal). **Andreas Fichtner:** conceptualization (equal), formal analysis (equal), funding acquisition (equal), software (equal), supervision (equal), visualisation (equal), writing – review and editing (equal). **Matthias Kunz:** data curation (supporting), writing – review and editing (supporting). **Michaela Hildebrand:** data curation (supporting), writing – review and editing (supporting). **Helge Bruelheide:** writing – review and editing (supporting). **Catherine Potvin:** writing – review and editing (supporting). **Florian Schnabel:** data curation (supporting), writing – review and editing (supporting). **Goddert von Oheimb:** conceptualization (equal), funding acquisition (equal), supervision (equal), writing – review and editing (equal).

## Funding

This work was supported by International Research Training Group TreeDi, supported jointly by the Deutsche Forschungsgemeinschaft (DFG, German Research Foundation) (Grant 319936945/GRK2324) and the University of Chinese Academy of Sciences (UCAS).

## Conflicts of Interest

The authors declare no conflicts of interest.

## Supporting information


**Figure S1.** ece373610‐sup‐0001‐Supinfo.zip.

## Data Availability

All data supporting this study are available at https://doi.org/10.5281/zenodo.18763142.
