# Supplementary material for: Canopy Definitions Shape Canopy Space Filling–Productivity Relationships: Evidence From Terrestrial Laser Scanning
Source: Ecol Evol. 2026 May 4;16(5):e73610. doi: 10.1002/ece3.73610 (PMC13139647; doi:10.1002/ece3.73610)

Supplementary Materials for

**Strength of the relationship between canopy space filling and stand productivity varies with different canopy definitions**

Tama Ray et al.

*Corresponding authors: tama.ray@mailbox.tu-dresden.de

This PDF file includes:

Figs. S1 to S2

Table S1


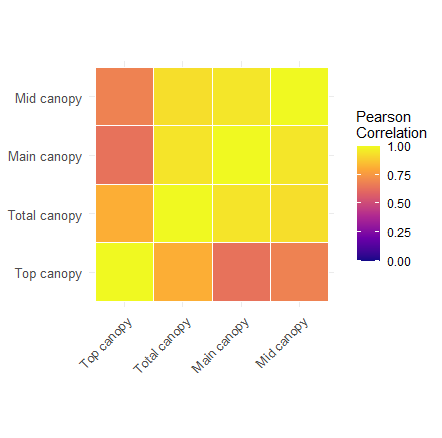


**Fig. S1 Correlation matrix between definitions of canopy space filling.**  The strength of the correlation is indicated by different colour shades (dark purple: neutral correlation, yellow: strong positive correlation).


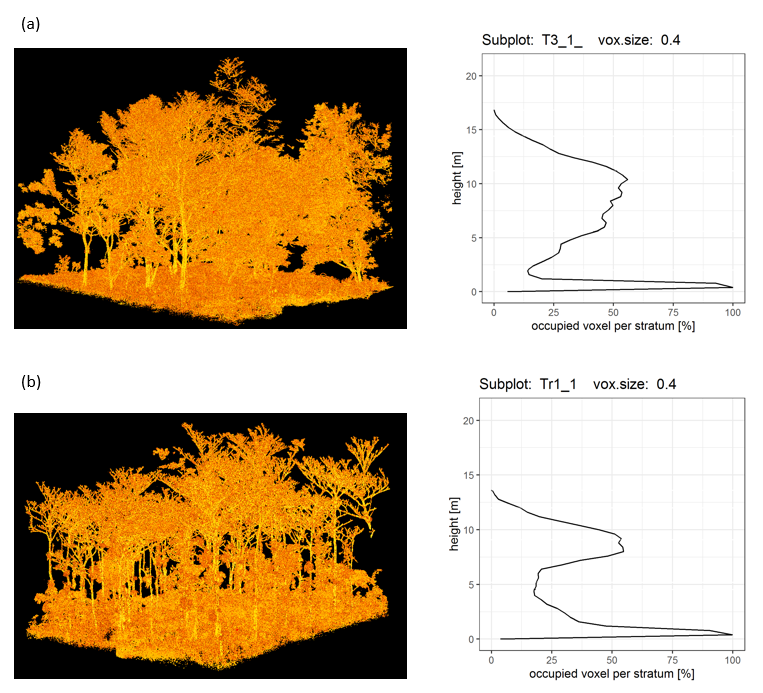


**Fig. S2 Representation of point cloud of subplots (left side) and canopy space filling per stratum through voxelisation approach of 0.4 voxel size (right side).** (a) indicates the three species mixture subplot while (b) monoculture subplot.

**Table S1 Results of generalised mixed-effect models for the effect of canopy space filling index (CSFI), mixture type (monocultures versus tree species-mixtures) and their interaction on stand productivity (annual wood productivity, AWP).** Chi-square (χ²) and *p*-values were obtained from likelihood ratio tests. Significant terms (*p* < 0.05) are highlighted in bold.


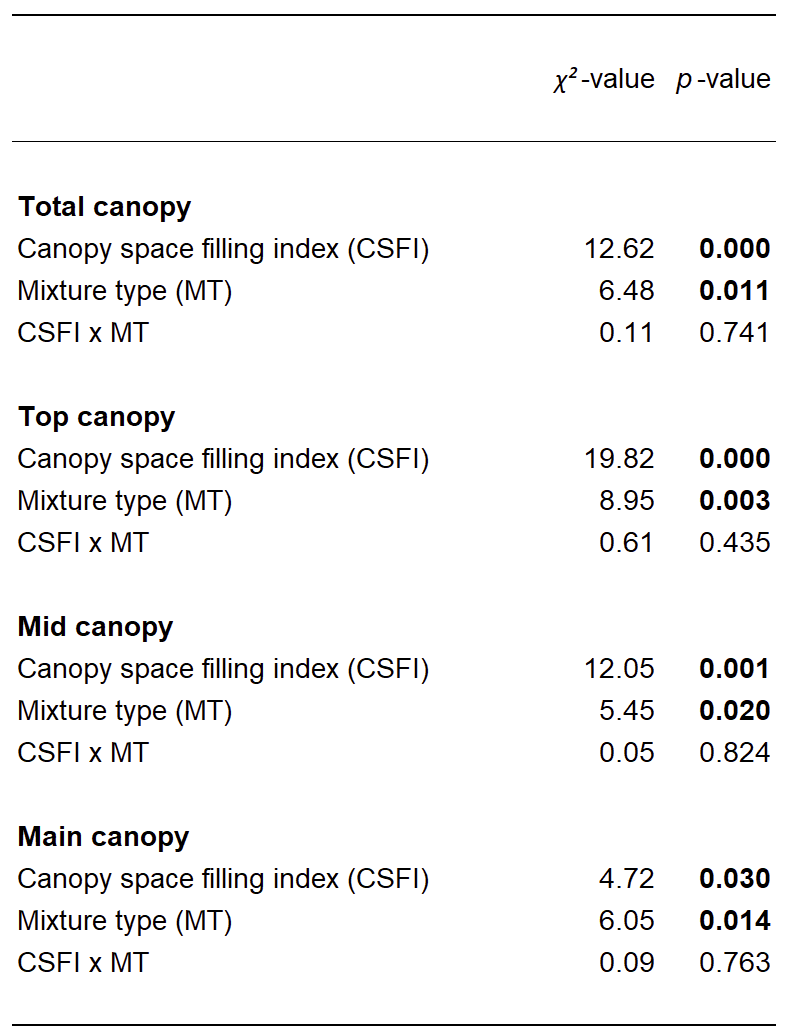

Supplement: Supplementary file 1 — Figure S1. ece373610‐sup‐0001‐Supinfo.zip. [file ECE3-16-e73610-s001.zip › Supplementary Materials.docx]
