# Supplementary figures and images for: Canopy Definitions Shape Canopy Space Filling–Productivity Relationships: Evidence From Terrestrial Laser Scanning
Source: Ecol Evol. 2026 May 4;16(5):e73610. doi: 10.1002/ece3.73610 (PMC13139647; doi:10.1002/ece3.73610)

(a)

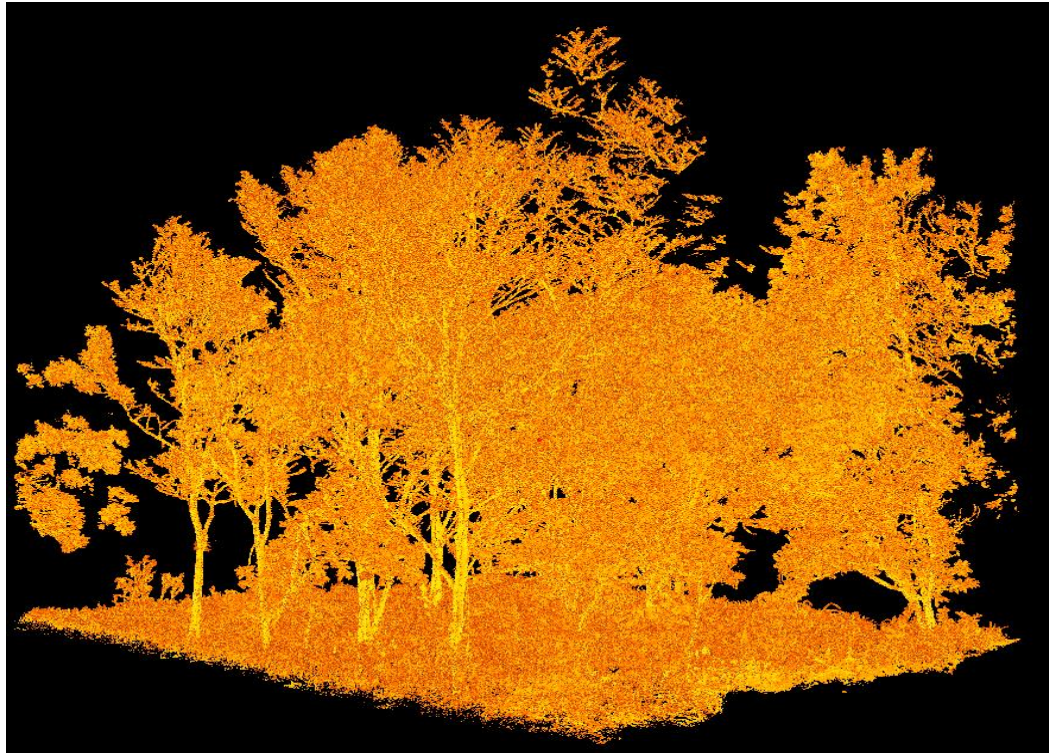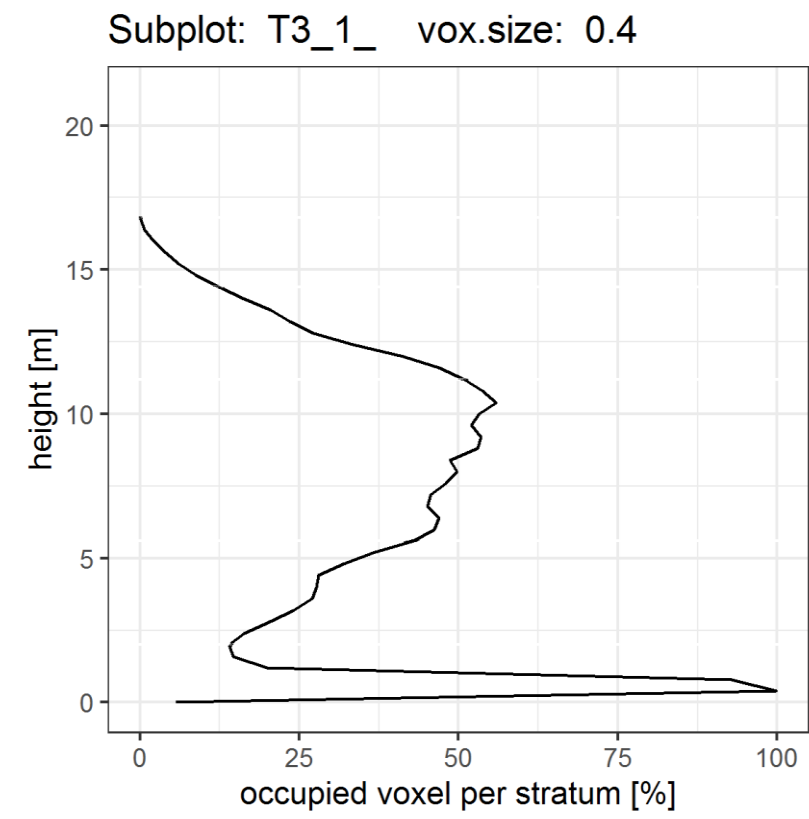

(b)

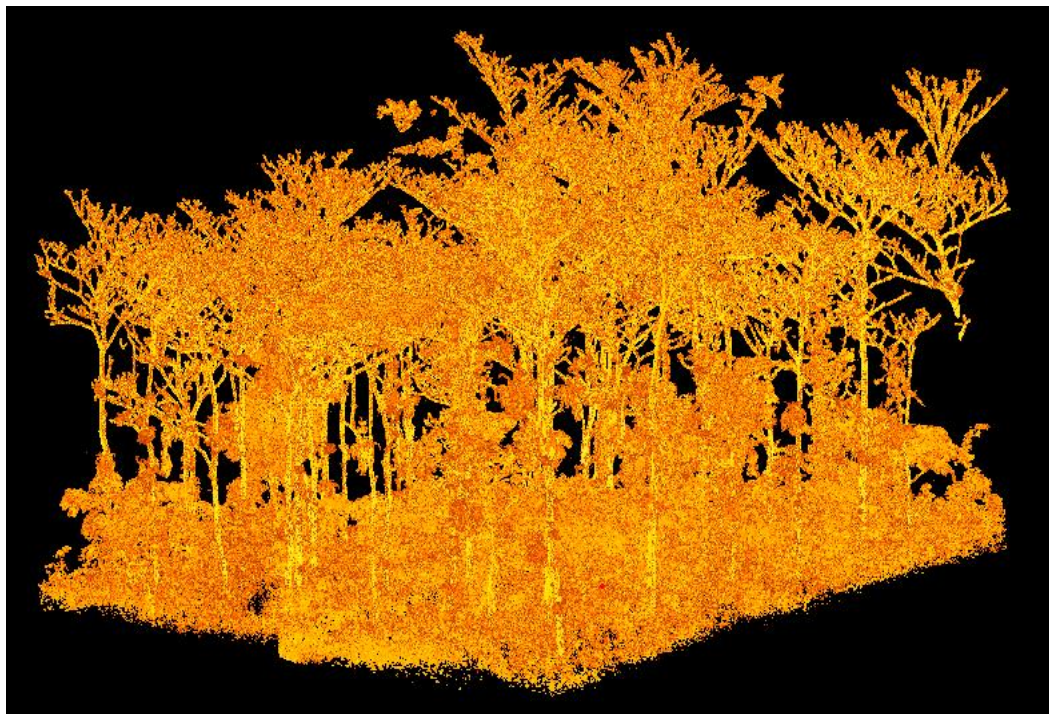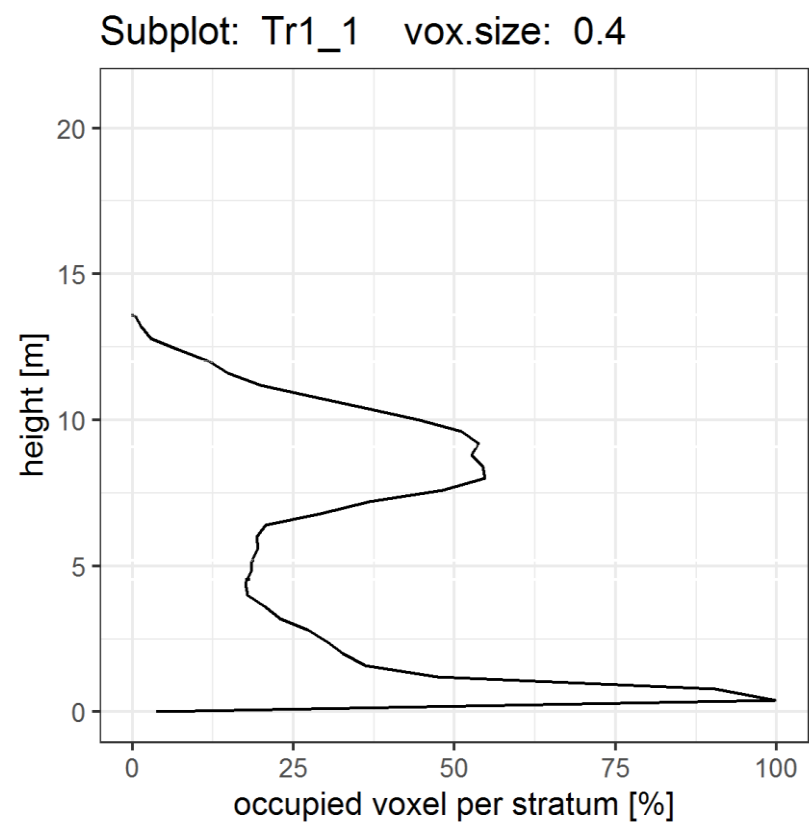

Supplement: Supplementary file 1 — Figure S1. ece373610‐sup‐0001‐Supinfo.zip. [file ECE3-16-e73610-s001.zip › ECE3_73610_f2_Figure_S2.pdf]

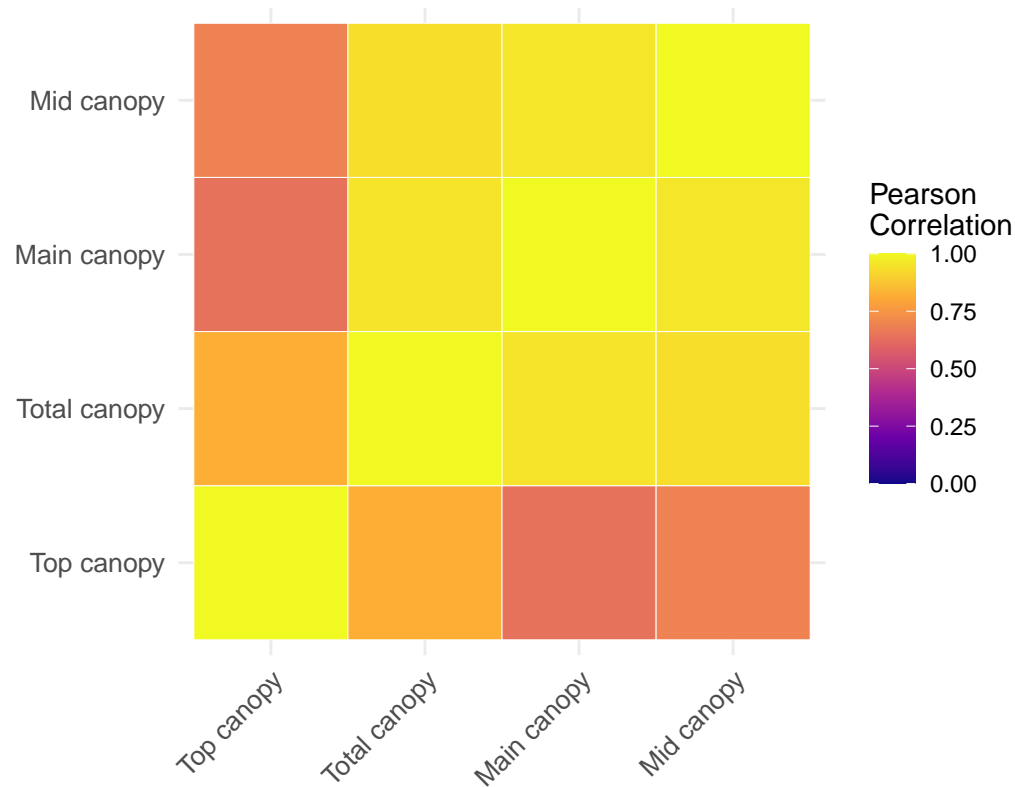

Supplement: Supplementary file 1 — Figure S1. ece373610‐sup‐0001‐Supinfo.zip. [file ECE3-16-e73610-s001.zip › ECE3_73610_f1_Figure_S1.pdf]
